# Phytochemical Profile and Analgesic Properties of Chicory Root Extract in the Hot-Plate Test in Mice

**DOI:** 10.3390/ijms26136387

**Published:** 2025-07-02

**Authors:** Łukasz Duda, Zbigniew Włodzimierz Pasieka, Monika Anna Olszewska, Magdalena Rutkowska, Grażyna Budryn, Andrzej Jaśkiewicz, Barbara Kłosińska, Karolina Czajkowska, Karol Kamil Kłosiński

**Affiliations:** 1 Department of Biomedicine and Experimental Surgery, Faculty of Medicine, Medical University of Lodz, Narutowicza 60, 90-136 Lodz, Poland; zbigniew.pasieka@umed.lodz.pl (Z.W.P.); barbara.klosinska@umed.lodz.pl (B.K.); kczajkowska2701@gmail.com (K.C.); 2 Department of Pharmacognosy, Faculty of Pharmacy, Medical University of Lodz, Muszynskiego 1, 90-151 Lodz, Poland; monika.olszewska@umed.lodz.pl (M.A.O.); magdalena.rutkowska@umed.lodz.pl (M.R.); 3 Institute of Food Technology and Analysis, Faculty of Biotechnology and Food Sciences, Lodz University of Technology, B. Stefanowskiego 2/22, 90-537 Lodz, Poland; grazyna.budryn@p.lodz.pl; 4 Department of Sugar Industry and Food Safety Management, Faculty of Biotechnology and Food Science, Lodz University of Technology, Wolczanska 171/173, 90-530 Lodz, Poland; andrzej.jaskiewicz@p.lodz.pl; 5 Biomaterials Research Laboratory, Faculty of Medicine, Medical University of Lodz, Narutowicza 60, 90-136 Lodz, Poland

**Keywords:** chicory extract, chicory root, sesquiterpenes, lactones, polyphenols, LC-MS, toxicity, analgesic properties, paracetamol

## Abstract

*Cichorium intybus* L. (common chicory) is a medicinal plant valued for health-promoting effects. Although analgesic properties are known for chicory sesquiterpenes, the effects of extracts need yet to be explored. This study aimed to evaluate for the first time the analgesic effect (against nociceptive pain) of the root extract from *C. intybus* var. *foliosum*. The target evaluation was preceded by toxicity tests in vivo and phytochemical standardization of root extracts prepared with different extraction methods—pectinase-assisted, pressure-assisted, and a combination of both—to choose the most effective one. The phytochemical profiling involved UHPLC-PDA-ESI-MS/MS and UHPLC-PDA analyses. The toxicity and the analgesic effects were tested in mice following the OECD 423 guideline and the hot-plate test, respectively. The highest recovery of bioactive compounds was achieved for the pressure-assisted extract: 642.5 mg sesquiterpene lactones, 187.1 mg phenolic acids, and 47.3 g inulin/100 g of dry matter. The extract showed no toxic effects at the oral dose of 2000 mg/kg body weight, including no histopathologic changes, in mice within two weeks (GHS Category 5/Uncategorized). The maximum analgesic effect (MAE) of the extract at 600 mg/kg was 6.75% for rearing and 13.7% for jumping, with the impact on the nocifensive reactions not differing significantly from those of paracetamol at 60 mg/kg. Despite the relatively low effects at 600 mg/kg, the verified safety and abundance of active compounds encourage further studies on the extract and its active fractions as potential approaches to complementary pain therapy, with special concern for their mechanisms of action.

## 1. Introduction

Humans have utilized various plants for dietary and medicinal purposes since time immemorial. This also applies to chicory (*Cichorium intybus* L., Asteraceae). Apart from being a source of food across the globe (leaves consumed in salads, roots used as a coffee substitute), different parts of this plant also play a significant role in modern phytotherapy [1,2]. The chicory root is listed in several pharmacopeias, including the current European Pharmacopoeia [3]. As recommended by the European Medicines Agency, chicory root can be used to relieve symptoms of mild digestive disorders (such as feelings of fullness, flatulence and slow digestion) and temporary loss of appetite [4]. A large body of experimental evidence, including some clinical trials, also demonstrates numerous other beneficial health effects of chicory, such as anti-inflammatory [5], antidiabetic [6], prebiotic [7], antioxidant [8], immunomodulating [9], hepatoprotective (seeds) [10], skin protecting [11], or even anticancer effects [12]. Apart from its application as a herbal medicine, chicory root is processed into flour and used as an ingredient in baked goods and coffee substitutes, products classified as functional food due to their antiviral, antimicrobial, hypolipemic, hypoglycemic, antioxidant, and cancer-preventing potential [13].

The medicinal and functional value of chicory root is connected to the abundant presence of bioactive compounds, primarily sesquiterpene lactones, phenolic acids, especially caffeoylquinic and caffeoyltartaric acids, and a polyfructan inulin [1,14]. Germacranolide-type sesquiterpene lactones, like lactucin and lactucopicrin, are the main bitter constituents, determining the choleretic, digestive and appetite-stimulating effects of chicory, synergistically with caffeic acid derivatives. These compounds are also potent anti-inflammatory agents, with their effectiveness verified in animal and clinical trials [5,15,16]. For instance, in patients with osteoarthritis, chicory root extract alleviated some disease symptoms, such as stiffness and inflammation-related pain, and increased global functional scores, showing at least 20% improvement in the defined response domains [5]. Interestingly, it was reported that sesquiterpene lactones lactucin, lactucopicrin, and 11,13-dihydrolactucin also have, similar to ibuprofen, antinociceptive and analgesic effects towards acute nociceptive pain in the hot-plate test in mice [17]. However, there is no information on such type of activity of chicory root extracts. Therefore, the present work is the first attempt to evaluate the potential antinociceptive effects of the root extracts of *C. intybus* var. *foliosum*, one of two main varieties of cultivated chicory [18].

Pain is an unpleasant sensation accompanying existing or impending tissue damage [19]. Three main types of pain are often difficult to distinguish between. The first type is nociceptive pain, characterized by a high pain threshold, occurring in the company of intense stimuli, such as touching something hot or sharp [20]. It is an acute pain, which can be described as an early-warning physiological protective system and is indispensable in detecting and reducing harmful stimuli. Immediate attention is then required, followed immediately by action through the withdrawal reflex [21].

The second type of pain, caused by the activation of the immune system through infection or injury, is referred to as inflammatory pain, while pain is one of the main characteristics of inflammation. Due to the increase in sensory sensitivity following tissue damage, this pain indirectly aids the healing process of the affected area by discouraging movement and physical contact. A hypersensitivity to pain in a given area, which is usually harmless, reduces the risk of greater damage and promotes recovery, e.g., with an inflamed joint or surgical wound after an operation or medical procedure. Although it is also protective and adaptive, it should be reduced in patients with inflammation [21].

Pain of the third type has no protective role and is maladaptive due to a malfunction of the nervous system. This pain, termed pathological, is not a symptom of a disorder but a condition of the nervous system, most often a disease state. It can occur during inflammation (dysfunctional pain) or damage to the nervous system (neuropathic pain). Pathological pain can thus be described as the result of amplified sensory signals in the central nervous system [21].

In all three cases, we are talking about a sensation referred to as pain. However, the different driving processes in each case require targeted treatment due to the separate mechanisms responsible for the condition [21]. In the case of surgery or other clinical procedures that involve noxious stimuli, the pain has to be suppressed, usually by opioids in specific doses or local anesthesia. We may also have to deal with the absence of this type of pain, which is a serious problem. Pain indifference or congenital insensitivity to pain most often leads to multiple injuries or even death. All this highlights the important protective role of pain [22]. Clinically, analgesics are often used to reduce pain, but care must be taken not to blunt nociceptive pain to a level disturbing its protective role [23,24].

Numerous studies have shed new light on the mechanisms and nature of pain, as well as the potential for developing new painkillers with better efficacy and fewer side effects [21]. Research has been increasingly directed toward natural sources of painkillers, with a focus on traditional plants that have favorable safety profiles [25]. One of these could be *C. intybus*, whose main active components—sesquiterpene lactones—have previously been shown to produce antinociceptive effects in animals [17].

The present study aimed to evaluate the analgesic effect (against nociceptive pain) of the root extract from *C. intybus* var. *foliosum* cultivated in Poland in an animal model. Prior to activity testing, three extracts obtained using different extraction techniques, i.e., pectinase-assisted, pressure-assisted, and a combination of both [18], were compared in terms of the recovery of three primary active fractions of chicory roots—sesquiterpene lactones, polyphenols, and polysaccharides (inulin). This approach allows us to evaluate the effect of extraction on the level of bioactive substances. The extract most promising for in vivo studies was selected based on comprehensive phytochemical profiling (qualitative and quantitative assays using ultra-high-performance liquid chromatography coupled with photodiode array detection and tandem mass spectrometry with electrospray ionization (UHPLC-PDA-ESI-MS/MS and UHPLC-PDA) after confirming its biocompatibility following the OECD 423 guideline “Acute oral toxicity—Acute toxic class method” [26] and classifying the extract to the appropriate Globally Harmonized System (GSH) category of health hazard. As the main focus of the study was on nociceptive pain, a hot-plate test in mice was eventually applied. This test refers to thermal pain (caused by exposure to high temperatures), which is nociceptive pain linked explicitly to nociceptors present in the skin. The efficacy of the chicory root extract was compared to that of the reference analgesic drug, paracetamol, and a placebo control.

## 2. Results and Discussion

### 2.1. Phytochemical Profiling

As one of the most valued industrial and medicinal plants, *C. intybus* is grown in numerous varieties. While most previous research focused on industrial chicory (*C. intybus* var. *sativum*) or did not specify which varieties/cultivars were analyzed, we decided to study the less explored root of *C. intybus* var. *foliosum* (vegetable chicory, Belgian endive). The first step of the study was a thorough chemical standardization of the chicory root extracts prepared using water and three extraction methods (pectinase-assisted, pressure-assisted, and a combination of both) as described in our previous paper [18]. Water was chosen as the extraction solvent due to its biocompatibility and suitability for further in vivo experiments in animals, where oral administration of an aqueous solution of tested preparations is required. Moreover, a water environment was crucial for applying the enzyme-assisted method.

As shown in Table 1, the qualitative UHPLC-PDA-ESI-MS/MS assay revealed the presence of several active constituents in the extracts, including sesquiterpene lactones (germacranolides, i.e., lactucin, lactucopicrin, and their derivatives), phenolic acids (caffeic acid derivatives, mostly monocaffeoylquinic acids and dicaffeoylquinic acids), and malic and quinic acids.

The identification was based on comparing the UV spectra, MS/MS fragmentation patterns and chromatographic data (retention times, elution order) of the analytes with those of authentic standards or literature data [27,28,29,30]. Except for 4-*O*-caffeoylquinic acid, all other compounds were present in all tested extracts; however, significant differences in their abundance were noticed between the extracts.

To evaluate the quantitative differences between the extracts and obtain insight into the impact of the extraction technique on the recovery of individual active constituents, a UHPLC-PDA analysis was performed. The results are shown in Table 2.

**Figure 1:**
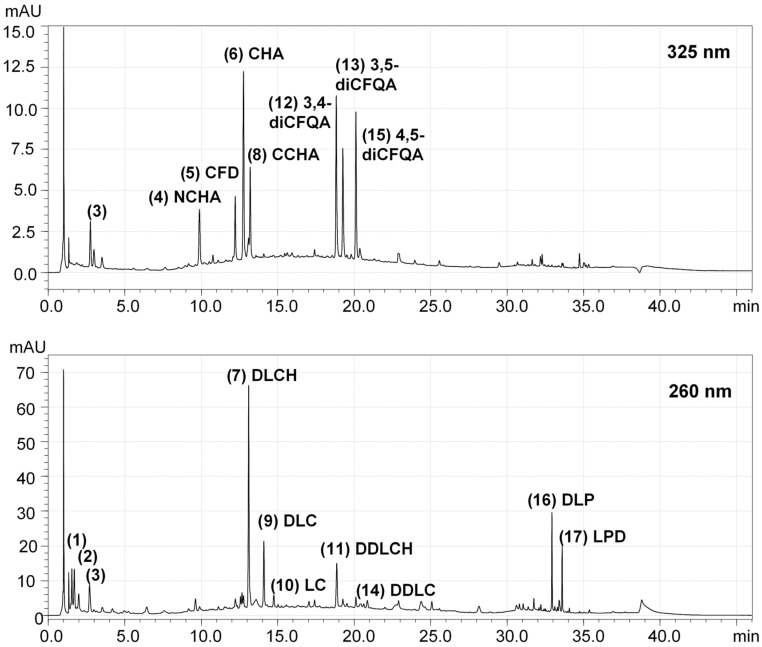
Representative UHPLC-PDA chromatograms at 325 nm and 260 nm of the chicory root extract obtained using pressure-assisted extraction (2nd method). The numbering of the compounds (1–17) corresponds to that in Table 1. Abbreviations: 3,4-diCFQA, 3,5-dicaffeoyl quinic acid; 3,5-diCFQA, 3,5-dicaffeoyl quinic acid; 4,5-diCFQA, 4,5-dicaffeoyl quinic acid; CCHA, cryptochlorogenic acid; CFD, caffeic acid derivative; CHA, chlorogenic acid; DDLC, 11(S),13-dihydro-8-deoxylactucin; DDLCH, 11(S),13-dihydro-8-deoxylactucin hexoside; DLC, 11(S),13-dihydrolactucin; DLCH, 11(S),13-dihydrolactucin hexoside; DLP, 11(S),13-dihydrolactucopicrin; LC, lactucin; LPD, lactucopicrin derivative; NCHA, neochlorogenic acid.

The extraction procedure impacted the quantitative composition of the extracts significantly, with phenolic acid pseudodepsides (mono- and dicaffeoylquinic acids) being most sensitive to different extraction conditions, primarily an acidic pH environment required for the enzyme-assisted extraction (Methods 1 and 3). In such conditions, the levels of pseudodepsides were noticeably reduced due to the hydrolysis of their ester bonds. Partial degradation of hydroxycinnamoyl esters in chicory root upon treatment with various enzymes has also been observed previously [31]. As for sesquiterpene lactones, their content under enzymatic conditions can either increase or decrease, depending on their molecular structure [31]. In the present study, the acidic hydrolysis of sesquiterpene glycosides, i.e., 11(S),13-dihydrolactucin hexoside (DLCH) and 11(S),13-dihydro-8-deoxylactucin hexoside (DDLCH), caused a decrease in their levels and a simultaneous increase in their free forms (aglycones), i.e., 11(S),13-dihydrolactucin (DLC) and 11(S),13-dihydro-8-deoxylactucin (DDLC), respectively. On the contrary, the levels of free sesquiterpenes not having their glycosidic analogs in the extracts, i.e., lactucin (LC) and 11(S),13-dihydrolactucopicrin (DLP), were reduced with the enzyme-assisted methods compared to the pressure-assisted procedure (Method 2). The representative chromatographic profile of the extract is shown in Figure 1.

A comparison of the results presented in Table 2 for different extraction techniques indicated that the hydrolytic impact of an acidic pH on phenolic acid esters and sesquiterpene glycosides in the enzyme-assisted methods had a lower influence on the total recovery of active compounds from chicory roots than the high-pressure-related enhancement in extraction efficiency. Consequently, the total content of sesquiterpene lactones was the highest in the chicory root extract prepared by pressure-assisted extraction, i.e., 642.5 mg/100 g dry matter (dm). This extract also has the highest total content of phenolic acids, reaching 187.1 mg/100 g dm (Table 2), and is inulin-abundant (47.3 g/100 g dm), following our previous study [18].

It is widely documented that high pressure significantly enhances the extraction efficiency of phytochemicals by reducing solvent viscosity and surface tension, disrupting cell membranes and increasing their permeability, thereby improving the permeation of the extraction solvent into the cells. In addition to the increased mass transfer, pressure-assisted extraction allows for reduced operating time, solvent amount, and thermal degradation of thermolabile constituents compared to traditional extraction techniques, such as heat reflux, soaking or Soxhlet extraction [32].

According to the literature, the qualitative and quantitative profiles of active phytochemicals in chicory may be significantly affected by numerous genetic, environmental and processing factors, including the cultivar, cultivation and climate conditions, storage time after harvesting, washing and cutting procedures, extraction solvents and extraction techniques, which may even reverse the proportions of primary compounds [28,33,34,35,36,37,38]. Nevertheless, the profile of individual sesquiterpene lactones and caffeoylquinic acids observed in our study is generally consistent with those previously reported for the same cultivar, *C. intybus* var. *foliosum*, especially in the case of the dominant constituents [28,31,39]. In all these studies and our investigations, lactucin derivatives (11(S),13-dihydrolactucin, 11(S),13-dihydro-8-deoxylactucin, and their glycosides) prevailed in the sesquiterpene fraction, followed by lactucopicrin derivatives; at the same time, chlorogenic acid and dicaffeoylquinic acid isomers dominated within polyphenols [28,30,31,39]. On the other hand, some previous studies have also detected trace amounts of dicaffeoyltartaric acids (cichoric acid isomers) and flavonoids in the alcoholic root extracts of chicory [30,39], which were absent in our aqueous preparations of chicory roots (Table 1 and Table 2). This fact can be attributed to their low solubility in water and limited occurrence in roots, as both types of these compounds are generally typical of aerial parts of chicory [30,39].

All the above-mentioned classes of chicory root components have been reported to have some analgesic properties in different types of pain, including nociceptive pain; however, sesquiterpene lactones appear most active in this direction [17,40]. It was shown that lactucin, lactucopicrin and 11,13-dihydrolactucin at doses of 30 mg/kg body weight (bw) exhibit analgesic effects in mice similar to ibuprofen (30 mg/kg), as recorded in two nociception models: hot-plate and tail-flick tests. Lactucopicrin was the most potent analgesic agent among the guaianolides tested; it was also effective at 15 mg/kg bw, and its activity was comparable to ibuprofen at most post-injection time points. Moreover, a reduction in the spontaneous locomotor activity of the animals was observed for lactucin and lactucopicrin, indicating their sedative properties [17].

The antihyperalgesic activity of chlorogenic acid was tested in the CCl-induced neuropathic pain in rats (chronic constrictive nerve injury) with two administration procedures: single intraperitoneal (i.p.) dose (50, 100, and 200 mg/kg bw) or chronic treatment (100 mg/kg/day, i.p., 14 days). The compound exhibited significant dose- and time-dependent antihyperalgesic effects [41]. For instance, the baseline mechanical paw withdrawal threshold was reduced by about 50% at 200 mg/kg bw at the time point of 15 min after injection and was comparable to that of gabapentin (100 mg/kg, i.p.); however, the effect of gabapentin lasted over 2 h, while that for chlorogenic acid lasted only about 45 min. Moreover, the chronic chlorogenic acid treatment prevented mechanical hyperalgesia development and attenuated CCI-induced histopathological changes, including fiber derangement, activation of neurological cells, and nerve fiber swelling [41]. In another study, local subcutaneous injection of chlorogenic acid (0.1–10 mM) into the peripheral receptive field suppressed the excitability of trigeminal spinal nucleus caudalis neurons (SpVc) in rats, with the effects comparable to that of lidocaine (37 mM) [42]. In addition, the known anti-inflammatory properties of chlorogenic acid (including inhibition of NF-κB signaling, MAPK, and PEG_2_) may be of service in relieving the inflammatory type of pain [43].

As for inulin, it is primarily tested as a prebiotic fiber and a biopolymer for drug delivery systems [44]. Its analgesic activity has not been tested yet; however, some anti-inflammatory effects in murine macrophages in vitro (downregulation of the NF-κB signaling pathway) were reported [45].

Therefore, based on the observed phytochemical profiles of three target chicory root preparations (Table 1 and Table 2), the extract obtained by the pressure-assisted technique (Method 2) was selected for further experiments in vivo. Apart from providing the highest recovery of sesquiterpene lactones and polyphenols, this method is distinguished by its technical simplicity, which facilitates its scalability and implementation in industrial conditions. With minimal technological requirements, the proposed procedure has significant potential to reduce operating costs in the industrial-scale production of an alternative drug or analgesic dietary supplement.

### 2.2. Toxicity In Vivo

**Figure 2:**
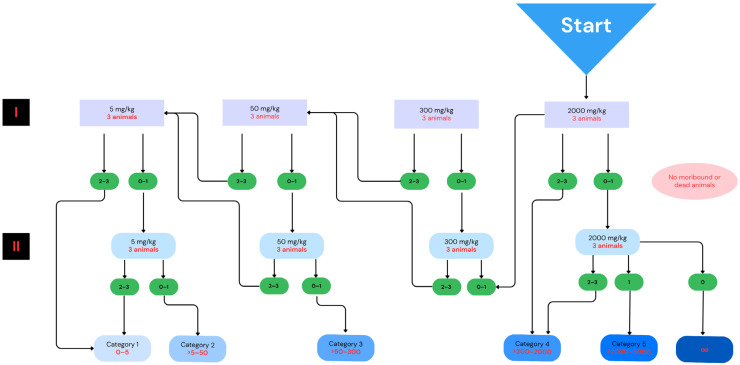
Classification scheme of acute oral toxicity tests applied for the chicory root extract following the OECD guideline [26,50]. “I” and “II” refer to the first and second stages of administration of the substance in accordance with OECD recommendations for toxicity studies. Due to no moribund or dead animals observed, the extract was tested at the dose of 2000 mg/kg bw only. The diagram was developed using the Canva software (Canva, Inc., Sydney, Australia; https://www.canva.com/ (accessed on 5 March 2025)).

Although chicory root extracts are commonly used for food and medicinal purposes, the products involving new extraction technologies, formulations, solvents, or delivery systems require verification of health hazards. Therefore, the selected extract was subjected to acute toxicity studies following the OECD guidance [26]. Analyzed substances should be administered in this test by the oral route at one of four fixed doses (5, 50, 300, or 2000 mg/kg) per test stage. The number of test stages depends on the mortality and/or moribund status of the experimental animals. Results allow the tested analytes to be ranked in a commonly used Globally Harmonized System (GSH) that classifies substances based on their hazard potential, with health hazards being one of the five main GHS categories [46]. Following these recommendations and considering the well-established use of aqueous chicory root extracts as food ingredients, as well as the accumulated experimental results of previous toxicity studies on the subject, proving their general safety [18,26,33,47,48,49], we decided to start the test from the highest dose of 2000 mg/kg bw (Figure 2). The extract obtained with the pressure-assisted method did not cause animal mortality during the observation of the mice over two weeks, nor did it show any toxic effects, such as behavioral abnormalities indicative of intoxication. The weight of the extract-treated animals was also maintained during the test period. Moreover, after euthanasia and dissection of the animals, no histopathologic changes indicating a possible toxic effect were found. All these observations allowed us to classify the extract to the GHS Category 5/Uncategorized, which confirmed its safety and allowed us to proceed to the next stage of the study for analgesic effects.

### 2.3. Hot-Plate Test

**Figure 3:**
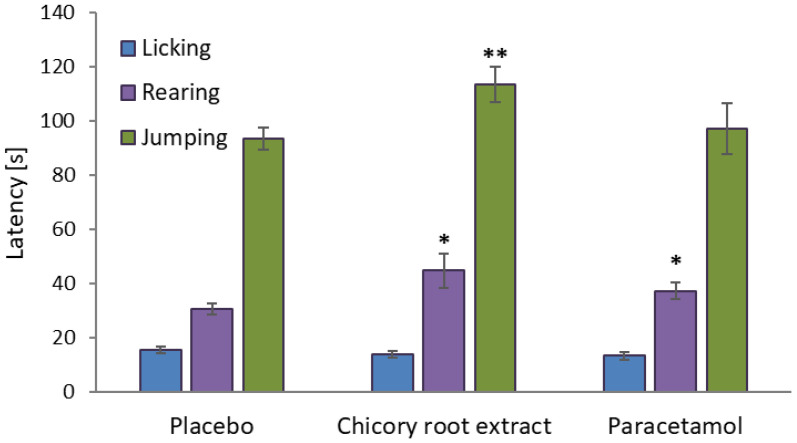
Differences in nocifensive reactions of mice in the hot plate test after treatment with the tested chicory root extract, reference drug, and placebo (means ± SEM, *n* = 8). Statistical significance of the test samples compared to placebo control in one-way ANOVA with a post hoc Dunnett test: ** *p* < 0.01, * *p* < 0.05. Error bars represent SEM values.

The potential antinociceptive and analgesic effects of the chicory root extract were analyzed in the hot-plate test by measuring a delay in the nocifensive reactions evoked by thermal stimuli in experimental rodents. This is a commonly used neurobehavioral test assessing the efficacy of various pain-relieving drugs [51]. The present study ran the tests in an acute mode (study completed in one day) by evaluating test samples after a single oral administration. The latencies in three common nociceptive pain behaviors of mice, such as paw licking, rearing, and jumping, were analyzed. Paracetamol, a commercially available non-opioid analgesic, was used as a reference drug at the dose of 60 mg/kg bw, while the extract was tested at 600 mg/kg bw. The dose was selected based on a literature review in which studies indicated significant analgesic activity of plant extracts at 600 mg/kg bw in the hot-plate and tail dip tests [52] and significant analgesic activity in the hot-plate and tail-flick models also at 600 mg/kg bw [53]. The dose of paracetamol was also selected based on a literature review; in the study, one of the doses in the hot-plate test was exactly 60 mg/kg bw [54], as was the case in the pain experiment, where 60 mg/kg bw was within the range of doses administered [55]. The selected doses were considered baseline values, within the range of commonly used doses of plant extracts and paracetamol. Results recorded for the particular pain symptom stages 30 min after administration of the tested substances are presented in Table 3 and Figure 3 as the latency in nocifensive reactions and the percentage of maximum analgesic effect (MAE), calculated from the following equation:

%MAE = (t_1_ − t_0_)/(t_2_ − t_0_) × 100 (1)

where

- t_0_ is the time from the placement of the mouse on the plate to the appearance of pain symptoms for placebo;
- t_1_ is the time from placement of the mouse on the plate to the onset of nocifensive reactions, for treatment;
- t_2_ is the maximum duration of the experiment (240 s).

**Table 3:** Latency to pain behavior and maximum analgesic effects (MAE) in the hot-plate test upon administration of the chicory root extract (600 mg/kg bw), paracetamol (60 mg/kg bw), and placebo control (vehicle) in mice.

	Licking		Rearing		Jumping	
	Latency (s)	MAE (%)	Latency (s)	MAE (%)	Latency (s)	MAE (%)
Chicory extract	13.8 ± 1.2 ^a^	nc	44.8 ± 6.3 ^a^	6.74	113.5 ± 6.4 ^a^	13.7
Paracetamol	13.3 ± 1.3 ^a^	nc	37.3 ± 3.2 ^a^	3.16	97.1 ± 9.5 ^a,b^	nc
Placebo	15.4 ± 1.2 ^a^	-	30.6 ± 2.0 ^b^	-	93.4 ± 4.0 ^b^	-

Latency results are presented as means ± SEM (*n* = 8). Different superscript letters (^a,b^) in one column indicate significant differences (*p* < 0.05) in Dunnett’s test. MAE values calculated from Equation (1). Nc, not calculated due to lack of statistical significance of the mean latency values compared to placebo control.

As indicated in Table 3 and Figure 3, administration of the chicory root extract at a dose of 600 mg/kg bw and paracetamol at 60 mg/kg bw produced a statistically significant elevation in the nociceptive threshold (*p* < 0.05), compared to the placebo control, in terms of rearing; moreover, the extract also delayed the nocifensive reactions of mice at the stage of jumping (*p* < 0.01). Considering the SEM data (Table 3), the effects of the extract were comparable to those of the drug.

In the first nociceptive behavior (licking), the extract and paracetamol did not show statistically significant effects (*p* > 0.05), which could be justified by stress, or the animals in the first stage were more sensitive to the appearance of the heat stimulus for reasons unrelated to treatment. Licking is also not a symptom that clearly indicates pain, but rather an irritation or a symptom that occurs in many other conditions, e.g., a foreign smell, cleaning, stress, or compulsive behavior. Moreover, licking is challenging to interpret because its intensity may vary depending on the individual characteristics of each animal [56,57]. The situation changed in the subsequent stages of nociceptive behavior (rearing, jumping). The chicory extract showed %MAE values of 6.74% for rearing and 13.7% for jumping, confirming its impact on the nociceptive pain response. The %MAE value for paracetamol was 3.16% for rearing (in other cases, %MAE cannot be calculated for the drug due to lack of statistical significance of the mean latency values compared to placebo control).

Collectively, all results obtained in the hot-plate test indicated that the chicory root extract might indeed have some analgesic potential, as was suggested earlier in the studies of pure active components of *C. intybus*—sesquiterpene lactones [17]. It should be noted that the working dose of the extract applied in the present study (600 mg/kg bw) corresponded to 3.86 mg/kg bw of sesquiterpene lactones (Table 2), which meant a 4–8-times lower dose than applied earlier (15–30 mg/kg bw). It also suggests that the low %MAE values observed in the groups receiving the extract may be related to the relatively low dose of the administered preparation and its complex composition, which involves both active sesquiterpene lactones and ballast substances. The effectiveness of the tested extract may be significantly potentiated by increasing the dose or concentrating the sesquiterpene fraction. Using more selective extraction solvents or purifying the extract from ballast substances by fractionated extraction or other separation techniques might be suitable for that purpose. Nevertheless, the effects observed for both the extract and paracetamol are relatively weak compared to the literature values reported for morphine—a reference analgesic agent exerting a central effect; the %MAE of morphine in the hot-plate test can be as high as 50%, depending on the dose and other test conditions [58,59]. On the other hand, the results obtained for paracetamol agree with its mechanism of action, which is more concerned with relieving inflammatory pain than central nociception.

Various types of sesquiterpenes and sesquiterpene-containing Asteraceae plants, including *C. intybus*, are often reported to have anti-inflammatory potential [60]. Previously, the effect of hydroalcoholic *C. intybus* root extract (CE, 300 and 500 mg/kg, administered daily for 7 days) was tested on lipopolysaccharide (LPS)-induced inflammation and pain in male mice. Intraperitoneal administration of LPS (0.83 mg/kg), utilized as an inflammatory pain-inducing agent, significantly decreased the response time of mice in the hot-plate test. Conversely, an extended response time was noted in animals treated with both concentrations of the plant extract (CE), compared to the LPS-treated control group. The results were also confirmed in the formalin test that produces a biphasic response: the early phase is thought to result from direct chemical activation of nociceptive afferent fibers, while peripheral inflammatory processes seem to be responsible for the late phase [61]. Eventually, both doses of CE significantly reduced licking and biting time in the second phase of the test, while only the increased dose of CE (500 mg/kg) was effective in the first phase, which suggested that the extract effects are a result of an anti-inflammatory response [62]. Similar conclusions have been drawn for the sesquiterpene lactone-containing extracts of *Achillea nobilis*. subsp. *neilreichii* [61] and *Artemisia ludoviciana* [63], investigated in the hot-plate, formalin, and tail-flick tests. All these findings, together with the well-established biological properties of compounds in the chicory root extract, discussed in Section 2.1, suggest that the modulation of inflammatory pain is worthy of further in-depth research as a therapeutic target for the extract. In upcoming studies, it is worth considering the use of purified fractions of active ingredients, as well as testing higher doses, which may increase the analgesic effectiveness of the extract.

## 3. Materials and Methods

### 3.1. Preparation of the Chicory Root Extract

The tested extracts were produced from the chicory root as described previously [18] with the extraction condition optimized earlier [64]. The plant material (root of *C. intybus* L. var. *foliosum*) was provided by the local chicory producer (Bakor Ltd., Skierniewice, Poland), washed with water and ground using a grinder with a 4 mm mesh diameter screen. For the extraction, three different protocols were applied (Methods 1–3) with the following technical principles for each method:

- Method 1: 5 kg of chicory roots, 8 L of water, 0.5 mL of 0.1% pectinase; pH adjusted to 4.0; maceration, 50 °C, 6 h;
- Method 2: 5 kg of chicory roots, 8 L of water; boiling in a pressure cooker, 0.2 MPa, 120 °C, 30 min;
- Method 3 (a combined method): 5 kg of chicory roots, 8 L of water, 0.5 mL of 0.1% pectinase; pH adjusted to 4.0; maceration at 50 °C for 6 h, and then boiling in a pressure cooker at 0.2 MPa, 120 °C, 30 min.

All extracts were filtered through a Büchner funnel, lyophilized using a Christ Delta 1–24 LSC freeze-dryer (Christ, Osterode am Harz, Germany), and stored at −25 °C until analysis.

The optimum pH for pectinase activity is in the range of 4.0–4.5. Although the enzyme can operate over a wider range from 3.0 to 6.0, it is in this optimum pH range that pectinase exhibits the greatest enzymatic activity, resulting in more efficient pectin breakdown. pH 4.0 was used in all extraction variants due to the fact that in an acidic environment (pH~4), even without enzyme activity, partial hydrolysis of pectin occurs, which increases cell wall permeability and facilitates the release of water-soluble compounds such as polyphenols. In addition, many plant compounds, including the aforementioned polyphenols, are more stable in an acidic environment. Also, the use of pectinase at pH 4.0 allows extraction to be carried out under milder conditions (lower temperature), which reduces thermal degradation of bioactive compounds, while increasing the extractability of bioactive compounds from chicory roots.

A pressure of 0.2 MPa for the extraction of chicory root components was chosen as a result of preliminary studies, as a compromise between extraction efficiency and preservation of the integrity of thermolabile compounds. At this pressure, the extraction temperature was about 120 °C, allowing a more efficient increase in the pressure gradient, solvent penetration into the cells, and better release of components, increasing the solubility of bioactive compounds and accelerating the diffusion of compounds into the liquid phase, thus reducing the extraction time to 30 min, while maintaining below the degradation thresholds of many phenolic compounds, thus maintaining the quality of the extract. Pressure at this level does not require specialized, expensive high-pressure equipment, making the process more economical and easier to implement in the food industry.

The combination of the two methods was designed to take advantage of the synergistic effect of enzymatic action (breakdown of pectic structures in cell walls) and pressure treatment (increased solvent penetration and release of intracellular compounds), leading to maximization of extraction efficiency while maintaining the quality of the extracts obtained [18].

### 3.2. Phytochemical Profiling of the Extracts

The qualitative UHPLC-PDA-ESI-MS/MS analysis was performed using a UHPLC-3000 RS system (Dionex, Dreieich, Germany) equipped with a diode array detector, an AmaZon SL ion trap mass spectrometer with an ESI interface (Bruker Daltonik, Bremen, Germany), and a Kinetex XB-C18 column (Phenomenex, Torrance, CA, USA). Separation conditions were as follows: 25 °C; flow rate 0.3 mL/min; solvent A (water/formic acid, 100:0.1, *v*/*v*); solvent B (acetonitrile/formic acid, 100:0.1, *v*/*v*); elution profile: 0–45 min 6–26% B (*v*/*v*), 45–55 min 26–95% B, 55–63 min 95% B, 63–70 min 95–6% B, 70–80 min 6% B (equilibration); scan from m/z 100 to 2200; ESI parameters: dry gas flow 9 L/min, dry temperature 300 °C; nebulizer pressure 40 psi; capillary voltage 4.5 kV.

The quantitative UHPLC-PDA analysis was performed using the Nexera X3 (Shimadzu, Kioto, Japan) system with an LC-40 pump, a photodiode array detector SPDM30A, and an ARION Polar C18 (2.2 μm, 2.1 mm × 100 mm) column. Separation conditions were as follows: 35 °C; flow rate 0.3 mL/min; injection volume 1 µL; solvent A (acetonitrile), solvent B (water/orthophosphoric acid, 99.5:0.5 *v*/*v*); elution profile: 0–3 min 3% A, 3–26 min 3–40% A, 26–30 min 40–90% A, 30–35 min 90% A, 35–36 min 90–3% A, 36–46 min 3% A (equilibration). Before injections, extract samples were dissolved in acetonitrile/water (7:3, *v*/*v*) to the concentration of 13 mg/mL and filtered (a PTFE syringe filter; 25 mm, 0.2 µm, VWR, Randor, PA, USA). UV-Vis spectra were recorded over the range of 200–600 nm. The peaks were identified based on the LC-MS/MS analysis, UV-Vis spectra, retention times, co-chromatography with authentic standards, and previous literature data on *C. intybus* and other species of the corresponding phytochemical profiles [30,65]. For quantitation purposes, the identified compounds were estimated as equivalents of HPLC-pure external standards (Sigma-Aldrich, Seelze, Germany/St. Louis, MO, USA): chlorogenic acid for monocaffeoylquinic acids (detection wavelength 325 nm), cynarin for dicaffeoylquinic acids (detection wavelength 325 nm), and lactucin for sesquiterpene lactones (detection wavelength 260 nm).

### 3.3. Toxicity Studies in Mice

The study aimed to evaluate the potential health hazards connected to the oral administration of the chicory root extract (obtained by the pressure-assisted extraction, Method 2) selected in the earlier stages of the research among three tested chicory extracts. All experiments in a mouse model were approved by the Local Ethics Committee for Animal Experiments at the Medical University of Lodz (No. 48/ŁB 215/2021, 4 October 2021). The toxicity tests followed OECD guideline 423 (“Acute Oral Toxicity—Acute Toxic Class Method” [26]. The study protocol, recommended by the OECD guideline, is presented in Figure 2.

The experiments were performed on female non-pregnant Balb/c albino mice (22–28 g, each). The tests included several steps: quarantine, handling, weighing, and labeling the experimental animals. They were housed in an experimental room in transparent polycarbonate cages, with three animals per cage, under controlled environmental conditions suitable for their species. Items enriching the animals’ environment (such as cardboard tubes) were placed in the cages. The research methods employed in the procedures were chosen to minimize or eliminate pain, suffering, distress, or the possibility of permanent bodily damage to the animals. The animals were provided with standard whole-animal feed and ad libitum access to drinking water.

The tested chicory extract (dissolved in deionized water) was administered per os at 2000 mg/kg bw (*n* = 3). A 1 mL syringe with an attached probe was used for the administration. The administered dose was the maximum dose included in the OECD 423 protocol. The dose was chosen at this level based on the results of previous toxicity studies of *C. intybus* root, both in vitro and in vivo, which indicated a low health hazard associated with chicory roots [18,26,33,47,48,49]. Moreover, the common alimentary use of chicory root also suggests that *C. intybus* is a low-risk plant, justifying the decision to start the study with the highest dose.

**Figure 4:**
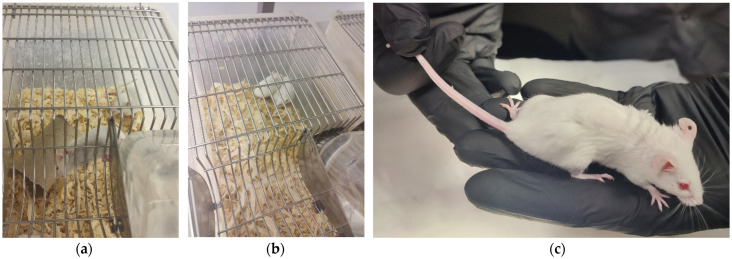
(**a**,**b**) Mice undergoing toxicity tests. (**c**) Mouse before euthanasia and dissection.

After dosing, each animal was placed in a separate cage (Figure 4) and observed at regular intervals: 30 min after administration, then hourly for 4 h, and subsequently once a day for the next 14 days. Observations included the condition of the skin, coat, eyes, mucous membranes, respiratory system, autonomic and nervous systems, somatomotor activity, and response to stimuli.

After this period, the animals were euthanized by an intraperitoneal administration of a lethal dose of sodium pentobarbital, followed by postmortem histopathological examination and macroscopic evaluation of the harvested organs. As neither mortality nor other toxic effects were observed for the animals exposed to the chicory root extract, the tests were terminated at the first stage, following the OECD guideline. Eventually, the extract-dependent health hazard was estimated to be in the appropriate category using the Globally Harmonized System (GSH) [46].

### 3.4. Hot-Plate Test in Mice

All experiments conformed with ethical guidelines for investigating experimental pain in conscious animals and were approved by the Local Ethics Committee for Animal Experiments of the Medical University of Lodz (No. 40/ŁB 243/2022, 12 September 2022). The experiments focused on the analgesic effect of the chicory root extract (obtained by pressure-assisted extraction, Method 2). For the study, the extract was dissolved in deionized water to a concentration of 100 mg/mL. Two controls were used in the experiment: a placebo control (saline, 0.9% sodium chloride in deionized water) and a reference analgesic agent (paracetamol, Sigma-Aldrich, Seelze, Germany/St. Louis, MO, USA).

The experiments were performed on 24 male Balb/c albino mice (22–28 g, each), divided into three groups of 8, where each group received a different substance. The tests included several steps, such as quarantine, habituation (taming), measurement of body weight, oral administration of the tested substance using a 1 mL syringe with an attached probe and testing the mice for nocifensive reactions upon thermal stimulus.

**Figure 5:**
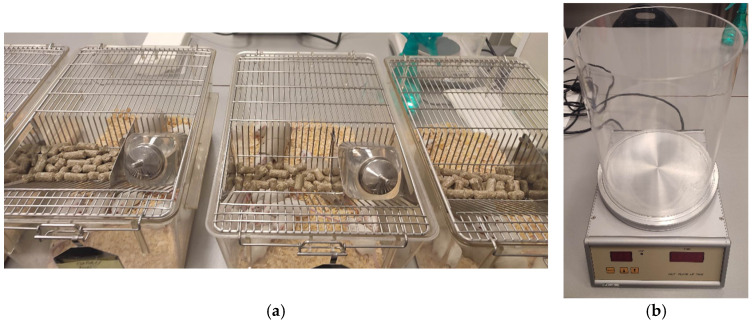
(**a**) Mice before the hot-plate test. (**b**) Laboratory analgesiometer (hot plate).

Analgesic properties were tested according to the method of Eddy and Leimbach [66,67,68]. Mice were placed on an aluminum plate of a thermal analgesiometer (LE7406, Harvard Apparatus, Holliston, MA, USA) maintained at 55 °C. A transparent plastic cylinder with a diameter of 14 cm and a height of 20 cm was used to restrict the movement of the animals on the plate (Figure 5).

Reaction time was recorded (when the animals licked their fore and hind paws, reared on their hind paws, and jumped) before and 30 min after administration of the extract (600 mg/kg bw), paracetamol (60 mg/kg bw), or placebo control. The experiment was stopped 240 s after the start to avoid soft tissue damage to the animals. After the hot-plate test, the mice were euthanized. Euthanasia was performed by rapid dislocation of the cervical vertebrae.

Initially, the tests involved 30 animals; however, during the analysis of behavioral data obtained from the hot-plate test, six animals (two from each group) were excluded from the analysis due to significant deviations from the normal behavior exhibited by the animals in their respective groups. These individuals were observed to exhibit, e.g., a lack of appropriate response to the stimulus or excessive sleepiness (suggesting a potential underlying disease), which could affect the reliability of the measurements. The exclusion aimed to maintain equal numbers and comparability of data between groups.

### 3.5. Statistics

The results were presented as means ± SD (standard deviation) or ± SEM (standard measurement error). The statistical significance of differences between the mean values was evaluated using a one-way ANOVA, followed by the post hoc Tukey test for multiple comparisons (for the phytochemical results) or post hoc Dunnett test (for the hot-plate test). Results with a *p*-value less than 0.05 were considered statistically significant. Satistica13.3Pl software (StatSoft Inc., Krakow, Poland) for Windows was used for all statistical analyses.

## 4. Conclusions

The combination of qualitative LC-MS/MS and quantitative UHPLC-PDA phytochemical profiling applied to three root extracts of *Cichorium intybus* L. *var. foliosum* represents an effective tool for selecting the extract most suitable for further experiments on the analgesic effects. The chosen pressure-assisted extraction method provided the highest recovery of the primary bioactive constituents of chicory roots, including sesquiterpene lactones, caffeoylquinic acids, and inulin. This method is distinguished by its simplicity, which facilitates its appropriate scaling and implementation in industrial conditions. With minimal technological requirements, the proposed procedure has the most significant potential to reduce operating costs in the long-term production of an alternative drug or analgesic supplement. The chicory root extract, produced using the selected method, was also proven to be safe in the acute oral toxicity test in mice at the high oral dose of 2000 mg/kg bw, which allowed its classification into GHS Category 5/Uncategorized [46].

Regarding the in vivo effectiveness, the extract at 600 mg/kg bw demonstrated some analgesic potential in the hot-plate test in mice, as evidenced by an increase in the nociceptive threshold compared to the placebo control. However, although the observed latency in nocifensive reactions of the extract-treated animals was comparable to the use of paracetamol at 60 mg/kg bw (*p* > 0.05), it was statistically significant (*p* < 0.05) only for later nociception signs, such as rearing and jumping. Moreover, the estimated maximum analgesic effect of the extract was relatively low, especially for the rearing behavior. Considering the preliminary nature of the study and its limitations (single-dose testing, focus on acute pain only), all these findings, along with the well-established anti-inflammatory properties of the extract constituents, primarily sesquiterpene lactones, suggest that further studies of the extract should focus on its impact on chronic inflammatory pain and testing a greater variety of doses and dose–response relationships. The entire mechanism of action of the extract in appropriate in vivo models also requires an in-depth evaluation. Moreover, it would be interesting to study whether the chicory root extract synergizes with paracetamol or other synthetic analgesics. In this context, future studies would likely benefit from using a concentrated fraction of sesquiterpenes to enhance the effectiveness of the tested extract.

## Figures and Tables

**Table 1:** Qualitative UHPLC-PDA-ESI-MS/MS identification of individual compounds in the chicory root extracts obtained by three extraction methods.

No.	Analyte	*R_t_* (min)	UV λ_max_ (nm)	Ionization Mode	m/z	MS^2^/MS^3^ Fragment Ions	Extract/Method
1	malic acid	1.7	210	[M–H]^−^	133	133 (100)	1, 2, 3
2	quinic acid	1.9	220, 270	[M–H]^−^	191	111 (100)	1, 2, 3
3	unknown	3.9	215, 300	[M–H]^−^	329	167 (100)	1, 2, 3
4	3-*O*-caffeoylquinic acid (NCHA, neochlorogenic acid) ^a^	6.7	216, 324	[M–H]^−^	353	191 (100), 179 (42), 135 (5)	1, 2, 3
5	caffeic acid derivative (CFD)	9.4	216, 325	[M–H]^−^	503	257 (100), 215 (34), 179 (15)	1, 2, 3
6	5-*O*-caffeoylquinic acid (chlorogenic acid, CHA) ^a^	11.2	216, 325	[M–H]^−^	353	191 (100)	1, 2, 3
7	11(S),13-dihydrolactucin acetyl-hexoside (DLCH)	12.0	258	[M–H]^−^	485	439 (100), 277 (3), 215 (16), 260 (12)	1, 2, 3
8	4-*O*-caffeoylquinic acid (CCHA, cryptochlorogenic acid) ^a^	12.6	216, 325	[M–H]^−^	353	191 (50), 179 (49), 173 (100)	2
9	11(S),13-dihydrolactucin (DLC)	14.3	258	[M–H]^+^	279	215 (100), 187 (27)	1, 2, 3
10	lactucin (LC) ^a^	16.9	258	[M–H]^+^	277	259 (40), 217 (100)	1, 2, 3
11	11(S),13-dihydro-8-deoxylactucin hexoside (DDLCH)	28.9	258	[M–H]^−^	469	261 (100), 217 (20)	
12	3,4-O-dicaffeoylquinic acid (3,4-diCAQA)	33.0	216, 325	[M–H]^−^	515	353 (100) ^b^, 299 (5), 203 (6) 191 (4), 173 (3), 191 (80) ^c^, 179 (60) ^c^, 173 (100) ^c^	1, 2, 3
13	3,5-O-dicaffeoylquinic acid (3,5-diCAQA)	33.5	216, 325	[M–H]^−^	515	353 (100) ^b^, 191 (3), 179 (3), 191 (100) ^c^, 179 (23) ^c^	1, 2, 3
14	11(S),13-dihydro-8-deoxylactucin (DDLC)	33.6	258	[M–H]^+^	263	244 (74), 217 (100)	1, 2, 3
15	4,5-O-dicaffeoylquinic acid (4,5-diCAQA)	36.6	216, 325	[M–H]^−^	515	353 (100) ^b^, 299 (3), 173 (3), 191 (20) ^c^, 179 (70) ^c^, 173 (100)	1, 2, 3
16	11(S),13-dihydrolactucopicrin (DLP)	50.0	258	[M–H]^+^	413	261 (100), 215 (20)	1, 2, 3
17	lactucopicrin derivative (LPD)	51.3	258	[M–H]^+^	439	411 (100), 277 (64)	1, 2, 3

^a^ Compounds identified with authentic standards. ^b^ MS^2^ fragment ions subjected to MS^3^ fragmentation. ^c^ MS^3^ fragment ions. R_t_, retention time; UV λ_max_, absorbance maxima in PDA spectra; [M–H]^−^, pseudomolecular ions in MS spectra recorded in a negative mode; [M–H]^+^, pseudomolecular ions in MS spectra recorded in a positive mode. Nomenclature of caffeoylquinic acid isomers is given according to IUPAC. For LC-MS chromatograms and detailed fragmentation patterns of detected compounds see Supplementary Materials Figures S1 and S2. Extract codes: 1, pectinase-assisted extraction; 2, pressure-assisted extraction; 3, combined pectinase–pressure-assisted extraction.

**Table 2:** The content of polyphenols and sesquiterpene lactones in chicory extracts obtained by three extraction methods (UHPLC-PDA analysis).

Constituent (mg/100 g dm)	Pectinase-Assisted Extraction (Method 1)	Pressure-Assisted Extraction (Method 2)	Pectinase + Pressure-Assisted Extraction (Method 3)
**Phenolic acids:**			
3-*O*-caffeoylquinic acid (NCHA)	<LOQ	16.83 ± 0.44 ^a^	4.79 ± 0.23 ^b^
caffeic acid derivative (CFD)	11.46 ± 0.08 ^b^	14.68 ± 0.17 ^a^	15.65 ± 0.73 ^a^
5-*O*-caffeoylquinic acid (CHA)	<LOQ	42.06 ± 1.01 ^a^	28.04 ± 1.37 ^b^
4-*O*-caffeoylquinic acid (CCHA)	<LOQ	20.52 ± 0.51 ^a^	6.10 ± 0.33 ^b^
3,4-dicaffeoylquinic acid (3,4-diCFQA)	<LOQ	35.63 ± 0.35 ^a^	8.66 ± 0.31 ^b^
3,5-dicaffeoylquinic acid (3,5-diCFQA)	<LOQ	26.22 ± 0.27 ^a^	21.27 ± 0.86 ^b^
4,5-dicaffeoylquinic acid (4,5-diCFQA)	<LOQ	31.14 ± 0.25 ^a^	8.56 ± 0.39 ^b^
Total phenolic acids (TPA)	**11.46 ± 0.08 ^c^**	**187.10 ± 3.01 ^a^**	**97.07 ± 4.23 ^b^**
**Sesquiterpene lactones:**			
11(S),13-dihydrolactucin acetyl-hexoside (DLCH)	44.39 ± 0.65 ^c^	270.74 ± 2.86 ^a^	87.46 ± 0.37 ^b^
11(S),13-dihydrolactucin (DLC)	156.00 ± 2.38 ^b^	97.25 ± 1.05 ^c^	190.37 ± 4.15 ^a^
lactucin (LC)	<LOQ	30.66 ± 0.25 ^a^	22.31 ± 1.08 ^b^
11(S),13-dihydro-8-deoxylactucin hexoside (DDLCH)	21.92 ± 0.55 ^c^	72.78 ± 0.45 ^a^	30.92 ± 0.50 ^b^
11(S),13-dihydro-8-deoxylactucin (DDLC)	61.47 ± 1.04 ^b^	22.62 ± 0.45 ^c^	83.59 ± 0.69 ^a^
11(S),13-dihydrolactucopicrin (DLP)	71.78 ± 2.81 ^b^	83.99 ± 3.74 ^a^	70.70 ± 2.35 ^b^
lactucopicrin derivative (LPD)	71.07 ± 2.02 ^a^	59.48 ± 2.23 ^b^	71.04 ± 3.72 ^a^
Total sesquiterpene lactones (TSL)	**426.63 ± 9.45 ^c^**	**642.52 ± 11.04 ^a^**	**556.39 ± 12.86 ^b^**

Results are presented as means ± SD (*n* = 6). Different superscript letters (^a–c^) in one row indicate significant differences (*p* < 0.05) in Tukey’s HSD test. Abbreviations: dm, dry matter; LOQ, limit of quantitation.

## Data Availability

The data presented in this study are available on request from the corresponding authors.

## Supplementary Materials

The supporting information can be downloaded at https://www.mdpi.com/article/10.3390/ijms26136387/s1.
