# Supplementary material for: Phytochemical Profile and Analgesic Properties of Chicory Root Extract in the Hot-Plate Test in Mice
Source: Int J Mol Sci. 2025 Jul 2;26(13):6387. doi: 10.3390/ijms26136387 (PMC12249640; doi:10.3390/ijms26136387)
Supplement: Supplementary file 1 [file ijms-26-06387-s001.zip › ijms-3703508-supplementary.pdf]

Supplementary Materials

Phytochemical Profile and Analgesic Properties of Chicory Root Extract in the Hot Plate Test in Mice

Łukasz Duda [1,*], Zbigniew Włodzimierz Pasieka [1], Monika Anna Olszewska [2], Magdalena Rutkowska [2], Grażyna Budryn [3], Andrzej Jaśkiewicz [4], Barbara Kłosińska [1], Karolina Czajkowska [1] and Karol Kamil Kłosiński [1,5,*]

1 Department of Biomedicine and Experimental Surgery, Faculty of Medicine, Medical University of Lodz, Narutowicza 60, 90-136 Lodz, Poland; zbigniew.pasieka@umed.lodz.pl (Z.W.P.); barbara.klosinska@umed.lodz.pl (B.K.); kczajkowska2701@gmail.com (K.C.)
2 Department of Pharmacognosy, Faculty of Pharmacy, Medical University of Lodz, Muszynskiego 1, 90-151 Lodz, Poland; monika.olszewska@umed.lodz.pl (M.A.O.); magdalena.rutkowska@umed.lodz.pl (M.R.)
3 Institute of Food Technology and Analysis, Faculty of Biotechnology and Food Sciences, Lodz University of Technology, B. Stefanowskiego 2/22, 90-537 Lodz, Poland, grazyna.budryn@p.lodz.pl
4 Department of Sugar Industry and Food Safety Management, Faculty of Biotechnology and Food Science, Lodz University of Technology, Wolczanska 171/173, 90-530 Lodz, Poland, andrzej.jaskiewicz@p.lodz.pl
5 Biomaterials Research Laboratory, Faculty of Medicine, Medical University of Lodz, Narutowicza 60, 90-136 Lodz, Poland
* Correspondence: karol.klosinski@umed.lodz.pl (K.K.K.); lukasz.duda@umed.lodz.pl (L.D.)

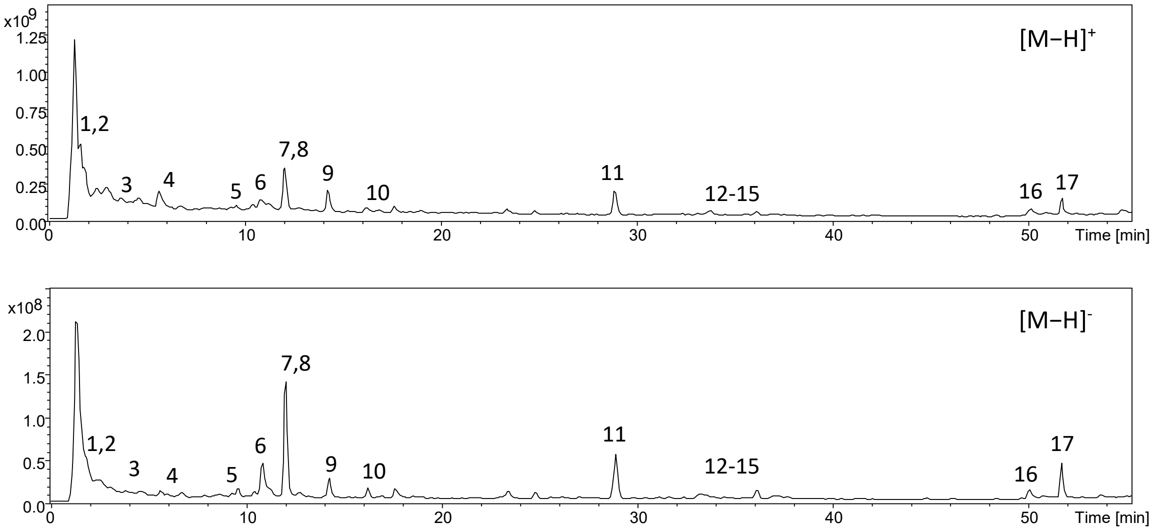

Figure S1. UHPLC-PDA-ESI-MS chromatograms of the chicory root extract obtained by the pressure-assisted extraction (Method 2) recorded in a positive mode [M-H]+ and a negative mode [M-H]-.

Compound 1 fragmentation

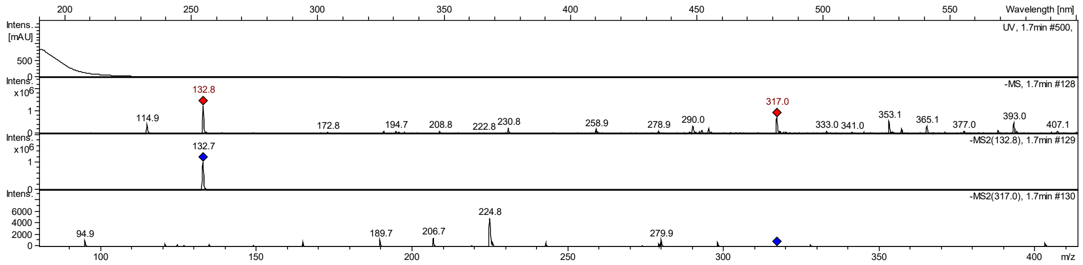

Compound 2 fragmentation

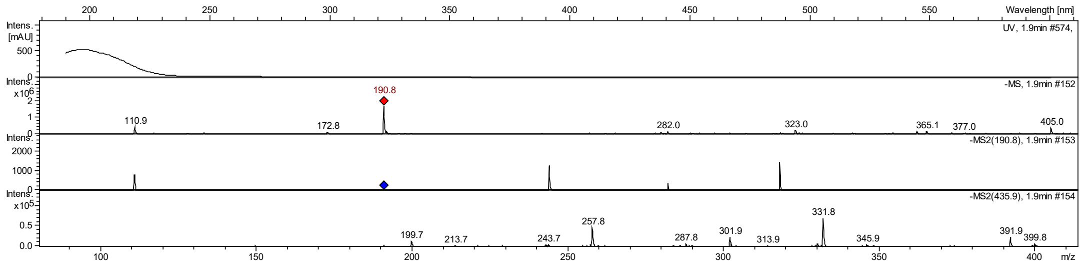

Compound 3 fragmentation

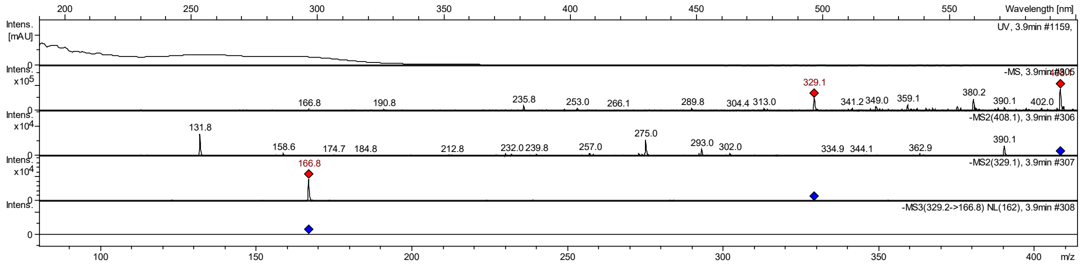

Compound 4 fragmentation

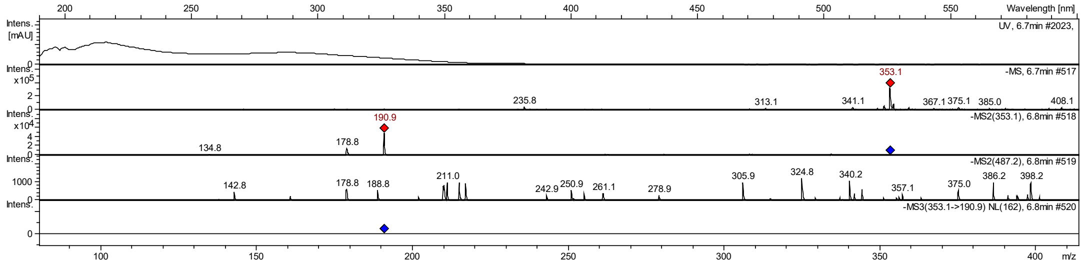

Compound 5 fragmentation

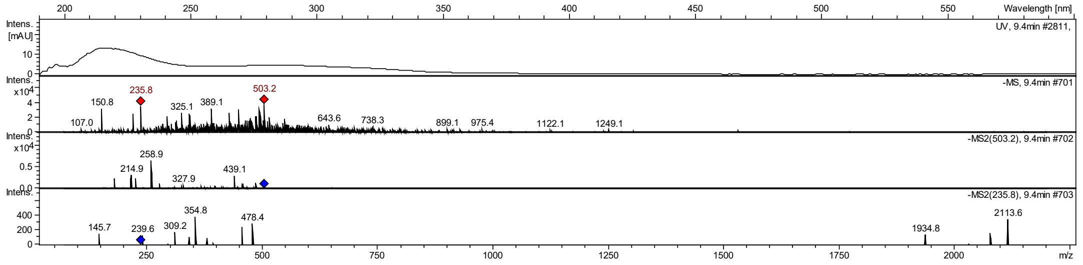

Compound 6 fragmentation

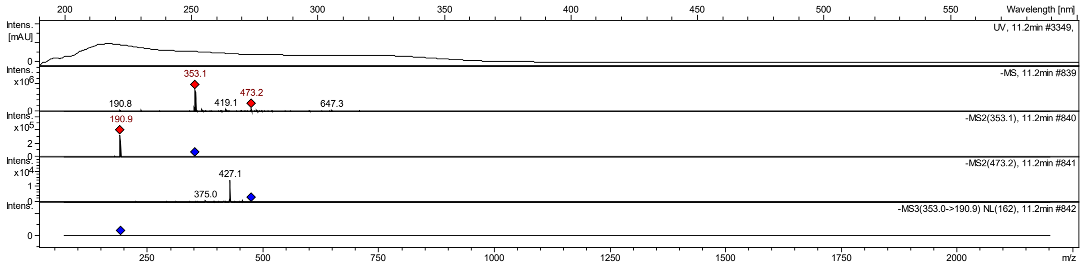

Compound 7 fragmentation

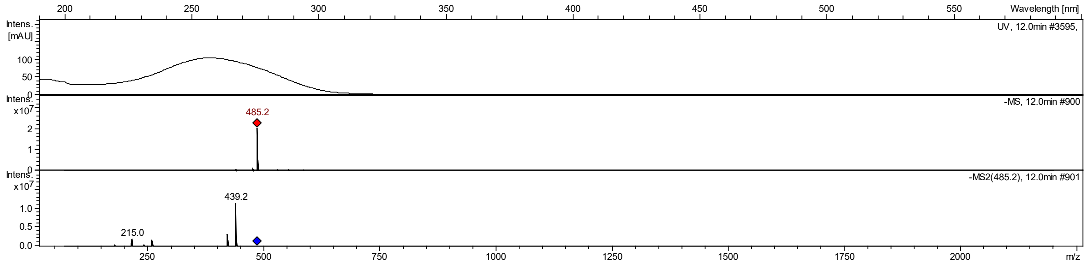

Compound 8 fragmentation

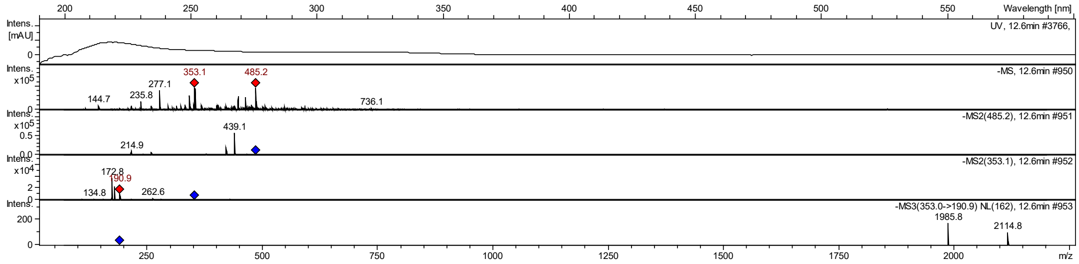

Compound 9 fragmentation

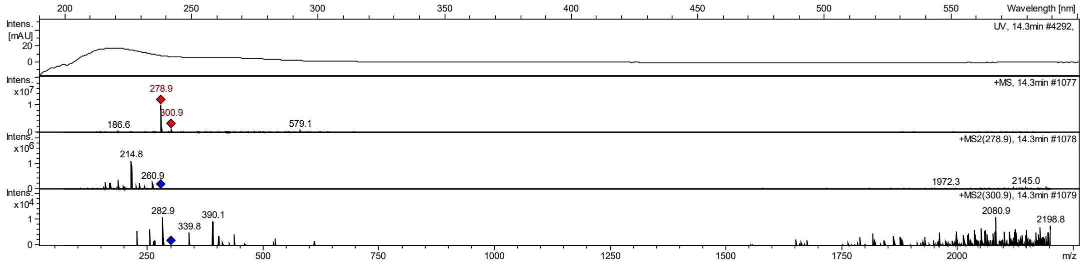

Compound 10 fragmentation

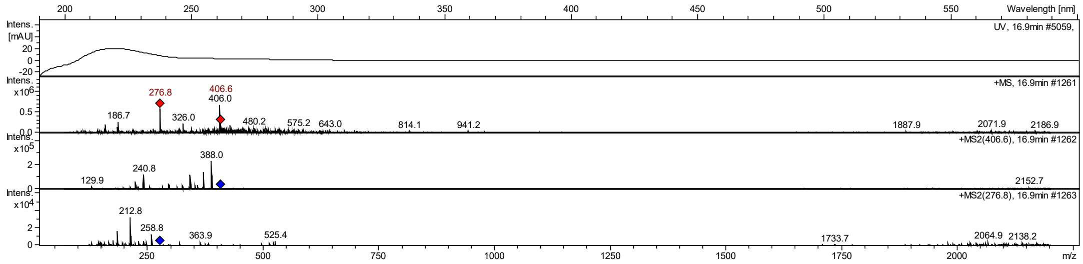

Compound 11 fragmentation

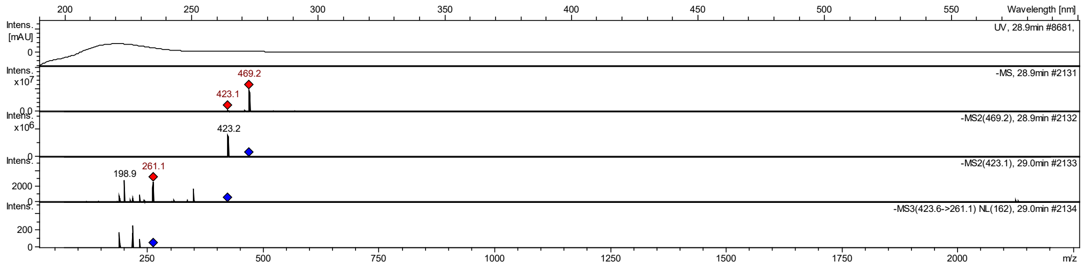

Compound 12 fragmentation

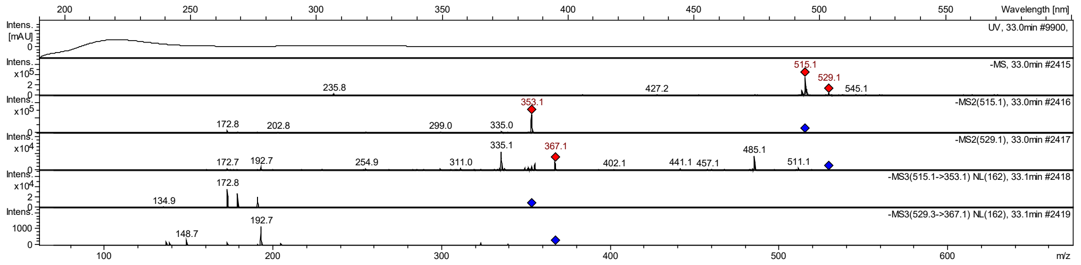

Compound 13 fragmentation

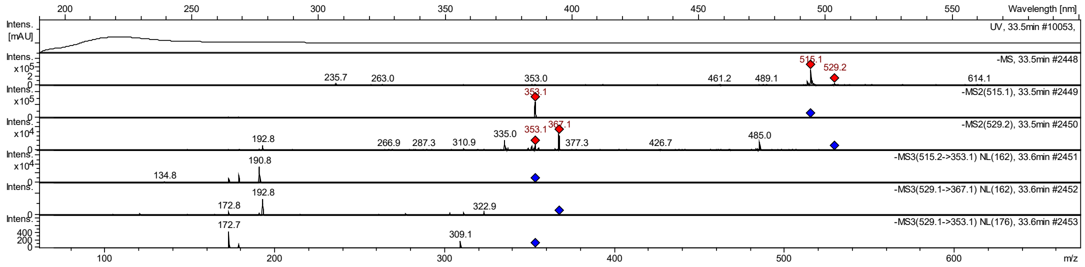

Compound 14 fragmentation

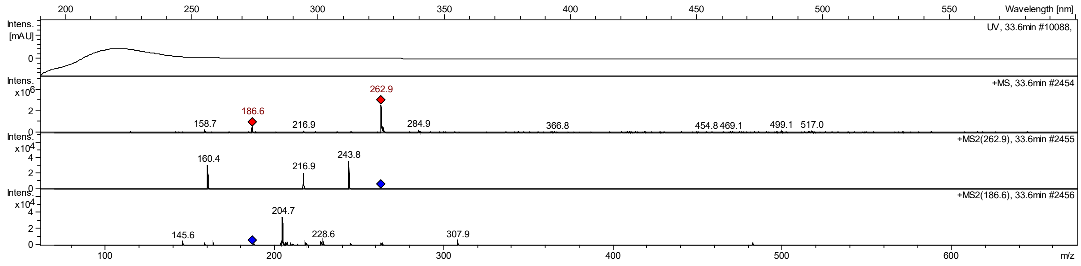

Compound 15 fragmentation

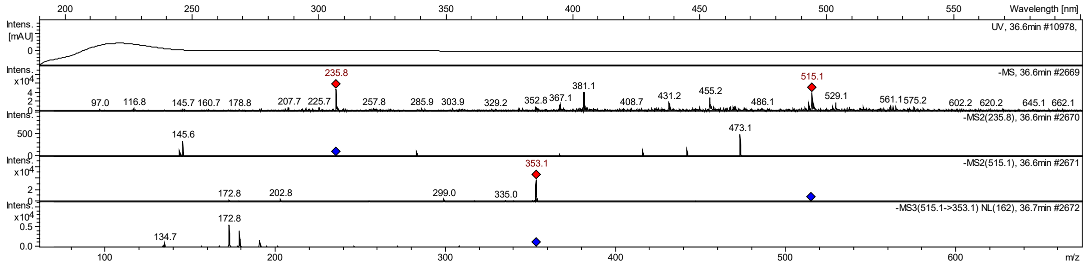

Compound 16 fragmentation

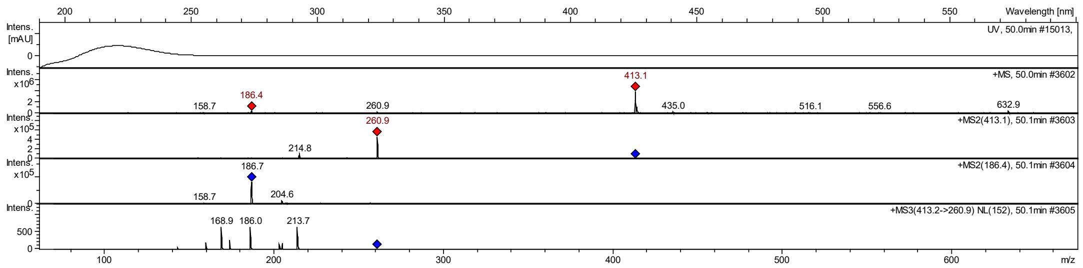

Compound 17 fragmentation

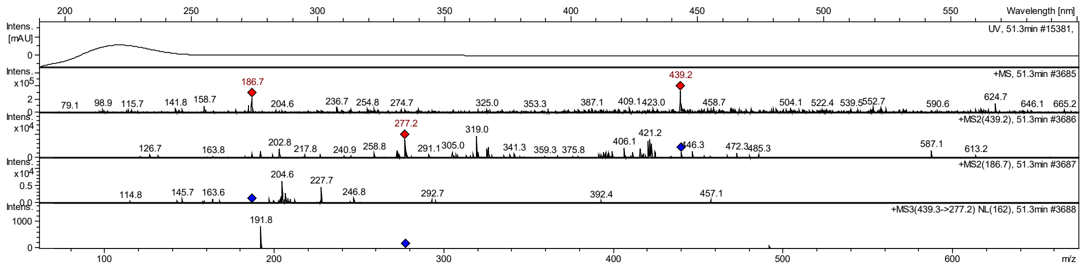

Figure S2. LC-MS/MS fragmentations of detected compounds. For detailed identification see Table 1 in the main article.
